# Enhanced music sensitivity in 9-month-old bilingual infants

**DOI:** 10.1007/s10339-016-0780-7

**Published:** 2016-11-05

**Authors:** Liquan Liu, René Kager

**Affiliations:** 1 School of Social Sciences and Psychology, Western Sydney University, Sydney, Australia; 2 Utrecht Institute of Linguistics OTS, Utrecht University, Utrecht, The Netherlands

**Keywords:** Infant, Language perception, Music perception, Bilingualism, Perceptual attunement, Acoustic sensitivity, Acoustic salience

## Abstract

This study explores the influence of bilingualism on the cognitive processing of language and music. Specifically, we investigate how infants learning a non-tone language perceive linguistic and musical pitch and how bilingualism affects cross-domain pitch perception. Dutch monolingual and bilingual infants of 8–9 months participated in the study. All infants had Dutch as one of the first languages. The other first languages, varying among bilingual families, were not tone or pitch accent languages. In two experiments, infants were tested on the discrimination of a lexical (*N* = 42) or a violin (*N* = 48) pitch contrast via a visual habituation paradigm. The two contrasts shared identical pitch contours but differed in timbre. Non-tone language learning infants did not discriminate the lexical contrast regardless of their ambient language environment. When perceiving the violin contrast, bilingual but not monolingual infants demonstrated robust discrimination. We attribute bilingual infants’ heightened sensitivity in the musical domain to the enhanced acoustic sensitivity stemming from a bilingual environment. The distinct perceptual patterns between language and music and the influence of acoustic salience on perception suggest processing diversion and association in the first year of life. Results indicate that the perception of music may entail both shared neural network with language processing, and unique neural network that is distinct from other cognitive functions.

## Introduction

Language and music are universal human faculties that involve high-level cognitive functions. While some linguists and biologists argue that the faculty of language is unique to humans (Hauser et al. 2002), infant music perception skills are often considered as a product of general perceptual mechanisms that are neither music- nor species-specific (Trehub and Hannon 2006). The intra- and inter-relationship between language and music perception in infancy has received less attention. Do young infants perceive the same pitch contrast differently when the pitch contours are embedded in language and in music? Does growing up in a bilingual environment alter infant language and music perception? This paper investigates 8- to 9-month-old infants’ perception of linguistic and musical contrasts differing in pitch, the (dis)association between the two cognitive functions, and how variations in the exposure of one domain may alter the perception of the other in the first year of life.

### The development of language and music in infancy

The perception of human speech and music is shaped by initial sensitivities at birth and later learning from the environment. Language-wise, infants are born with the ability to discriminate a wide range of native and non-native sound contrasts at birth. In the first year of life, infant sensitivity shifts towards the native language. This tuning in process is often referred to as perceptual attunement (e.g. Werker and Tees 1984; Kuhl et al. 1992). The perceptual attunement of lexical pitch occurs between 4 and 9 months (Mattock and Burnham 2006). Non-tone language learning infants, sensitive to lexical pitch contrasts at birth (Nazzi et al. 1998), no longer discriminate most lexical pitch contrasts 9 months after birth (Harrison 2000; Mattock et al. 2008; Yeung et al. 2013). This perceptual pattern is not absolute, since perceptual attunement is considered to be an “optimal” rather than a “critical”, clear-cut process (Werker and Tees 2005). Non-tone language learning infants retain sensitivity to acoustically salient lexical pitch contrasts even after 9 months of age (Liu and Kager 2014), a pattern that extends to adulthood (Chen et al. 2015), reflecting the influence of acoustic salience on lexical pitch perception.

Just as language, infants show initial sensitivity to music. After hearing repetitions of the original musical tone notes, infants of 5–10 months after birth are able to detect changes of single notes (Trehub et al. 1985) and of internal reordering of multiple notes (Trehub et al. 1984). Although infants, young children, and adults appear to perceive novel melodies in fundamentally similar ways on a neural level (Trehub et al. 1997), perceptual attunement presents itself in music. From a melody of ten notes, 8-month-old infants can detect a one note change in both diatonic (observing dominant harmony) and non-diatonic (out of key) conditions. Nevertheless, adult listeners fail consistently at the discrimination of diatonic changes (Trainor and Trehub 1992). Similar to language acquisition, one’s “native” music, the music representation forms carried by culture, is acquired through time and exposure. When testing participants growing up in a Western culture across ages, infants discriminate melodies from both Western musical conventions and unconventional musical chords. Children can differentiate the melodies more easily when they conform to the Western musical conventions compared to unconventional music. Adults are only able to discriminate the melodies in a conventional context (Lynch and Eilers 1992; Schellenberg and Trehub 1999). The perceptual narrowing of the conventionality effect from infancy to adulthood indicates the attunement of musical perception as the result of musical experience. In brief, infants follow similar perceptual attunement trajectories and form degrees of perceptual development across linguistic and musical domains in the first year after birth.

### The bilingual influence in infancy

Infants have an amazing capacity to adjust and adapt to their environment. Simultaneous bilingual infants hear virtually half of the input in each of their languages compared to monolinguals and become fluent speakers of two languages nonetheless (Gauthier and Genesee 2011). They pass major linguistic milestones approximately at the same ages as their monolingual peers (Werker 2012). Bilingual infants display general robust discrimination of the speech-sound distinctions in their native languages by the end of the first year of life (Bosch and Sebastián-Gallés 2003; Burns et al. 2007; Albareda-Castellot et al. 2011), and form stabilized perceptual patterns to native sounds by the second year after birth, at least for their dominant language (Dietrich et al. 2007; Liu and Kager 2015a).

At 8–9 months after birth, infant perception is not often stable. While some studies report the same pace of language development between monolingual and bilingual infants (Burns et al. 2007; Sundara et al. 2008; Albareda-Castello et al. 2011; Sundara and Scutellaro 2011), many others show a (temporary) delay in native language perception (Bosch and Sebastián-Gallés 2001, 2003; Sebastián-Gallés and Bosch 2009; Garcia-Sierra et al. 2011). Recent findings reveal a new, acceleration pattern in bilingual speech perception at 8–9 months, adding pieces to the existing puzzle: in the first year of life, bilingual Dutch infants exceeded their monolingual peers when discriminating native and non-native contrasts (Liu and Kager 2015b, 2016a).

Bilingual adaptation in language leads to perception and processing patterns distinct from those found in monolingual infants. These differences influence infants’ development across linguistic, cognitive and social domains (Kovács and Mehler 2009a, b; Kuhl et al. 2008; Shafer et al. 2011; Petitto et al. 2012; Kuipers and Thierry 2012; 2013; Brito and Barr 2012, 2014). The influence of bilingualism on infant music perception remains unclear. This paper is among the first to explore the influence of bilingualism on the cognitive processing of language and music.

### Research questions

When examining the relation between music and language, the processing of pitch has always been a main focus. This is not surprising since pitch is the most salient aspect for infants in both language (i.e. infant-directed speech, Fernald 1991) and music (Chang and Trehub 1977; Trehub et al. 1984). Accurate perception of the fine-grained difference between two pitches is a prerequisite for efficient music perception. The processing of musical melodies requires accurate detection of pitch changes as small as one semitone for both tone and non-tone language listeners (McDermott and Oxenham 2008). In the current study, pitch (fundamental frequency, F0) is kept constant in the stimuli across experiments, and we examine the different media by which pitch is delivered.

The research questions of the current study are: (1) what are non-tone language learning infants’ specific perceptual mechanisms for linguistic and musical pitch? (2) Does growing up in a bilingual environment alter infants’ perception of language and music? To answer these research questions, Dutch monolingual and bilingual infants were tested on lexical and violin pitch contrasts, respectively, in two experiments.

## Experiment 1

### Participants

A total of 42 Dutch monolingual and bilingual infants aged 9 months participated in the experiment. All bilingual infants were acquiring Dutch as one of their native languages, and the other language varied across participants (see Appendix). The degree of exposure to the non-dominant language was no less than 20% via a Multilingual Infant Language Questionnaire (Liu and Kager 2016). The mean (standard deviation, SD) degree of exposure to Dutch was 55% (17%) for bilingual infants. All parents reported normal hearing, no exposure to a tone language, and no excessive (more than 2 h per day) music exposure at home for their children. No parent worked as a musician as her/his profession. Eventually, data from 36 participants were included for analysis, with 18 participants per language background (mean age (SD): 268 (12) days; 64% males). Data from six participants were excluded from analyses for the following reasons: fussiness (3), program error during the experiment (1), and looking time difference exceeding 2 SD from the mean (2).

### Stimuli

**Fig. 1:**
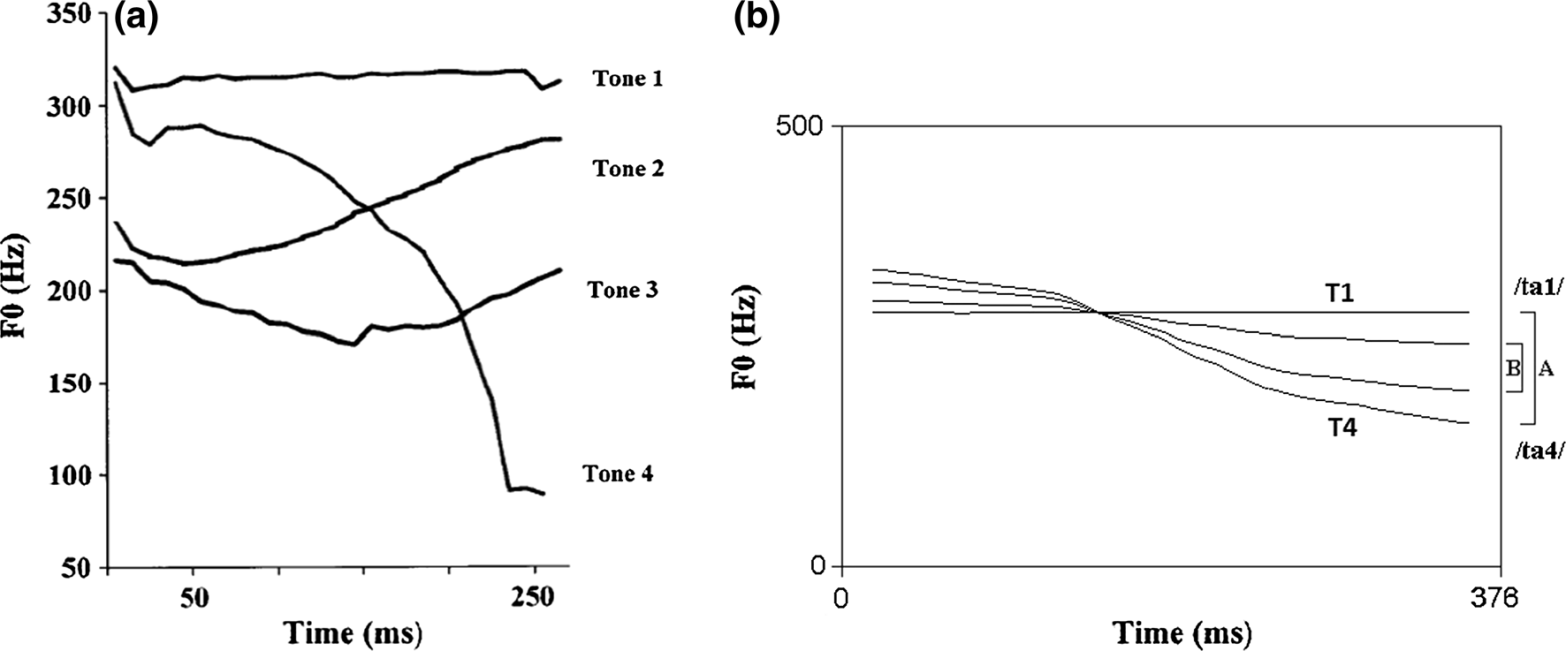
**a** Tones in Mandarin Chinese (*Source* Wang et al. 2001) *left*. **b** Pitch contours of the contracted T1–T4 [B] contrast created from T1–T4 [A] and adopted in the current study to reduce contrast acoustic salience *right*

**Fig. 2:**
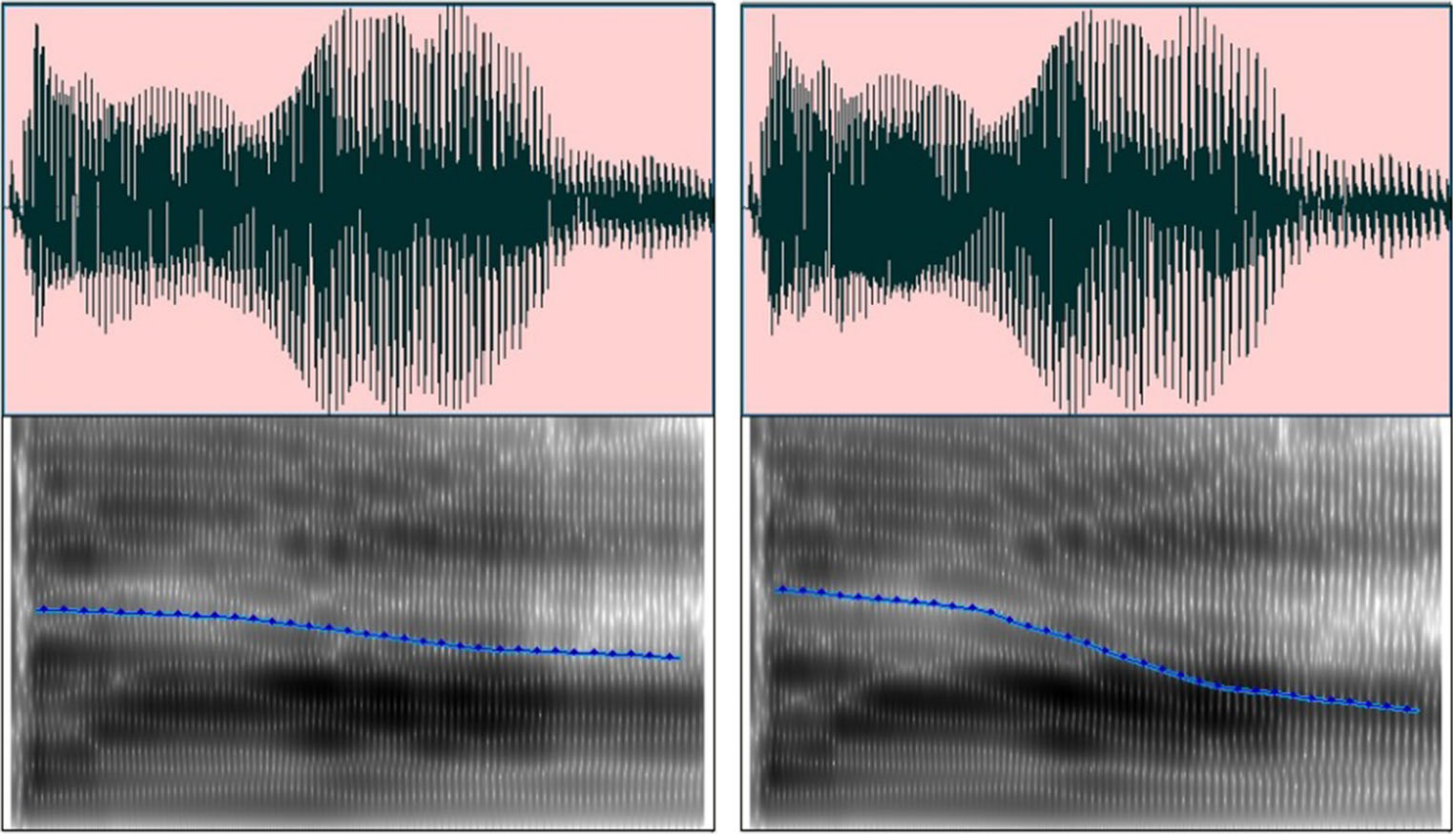
Oscillograms and spectrograms for the contracted T1 (*left*) and T4 (*right*)

Salient pitch contrasts can be detected by non-tone language listeners across ages (Liu and Kager 2014; Hallé et al. 2004). We reduced acoustic salience by manipulating F0 in the current study. Four lexical tones exist in Mandarin Chinese (Fig. 1a): high-level (T1), middle-rising (T2), low-dipping (T3), and high-falling (T4). The tone-bearing syllable was /ta/. Both /ta1/ “build” and /ta4/ “big” are legal words in Mandarin Chinese. The vocalisations of a Chinese female speaker were recorded using the computer program Audacity^1^ via a Genelec 1029A Active Speaker system in a soundproof booth in the phonetics laboratory of [name suppressed] University. Four natural T1–T4 pairs were recorded. To avoid a ceiling effect due to the high acoustic salience of the T1–T4 contrast (Huang and Johnson 2010; Sun and Huang 2012; Liu and Kager 2014), an acoustically contracted contrast was created from a T1–T4 tonal contrast by manipulating the F0 direction via the software PRAAT (Boersma and Weenink 2010) to reduce the acoustic salience of the contrast. Four interpolation points along the pitch contours (at 0, 33, 67, and 100%) were introduced. The F0 values occurring at 3/8 and 3/4 of the pitch distance of the original T1–T4 contrast were calculated at these interpolation points. Two new pitch contours were generated linking these points. The contracted contrast (Fig. 1b, contrast B) shares similar acoustic properties with the T1–T4 contrast (Fig. 1b, contrast A), except for featuring a narrower distance between the pitch contours, thus shrinking the perceptual distance between the two tokens (Fig. 2). Four pairs of the contracted contrast were generated to account for within-speaker variation. Five native speakers of Mandarin Chinese listened to the stimuli in the environmental settings and judged that the stimuli sounded natural. Since non-tone language learning infants experience perceptual attunement of lexical pitch at 9 months of age, they should be less sensitive to the contrast.

### Procedure

**Fig. 3:**
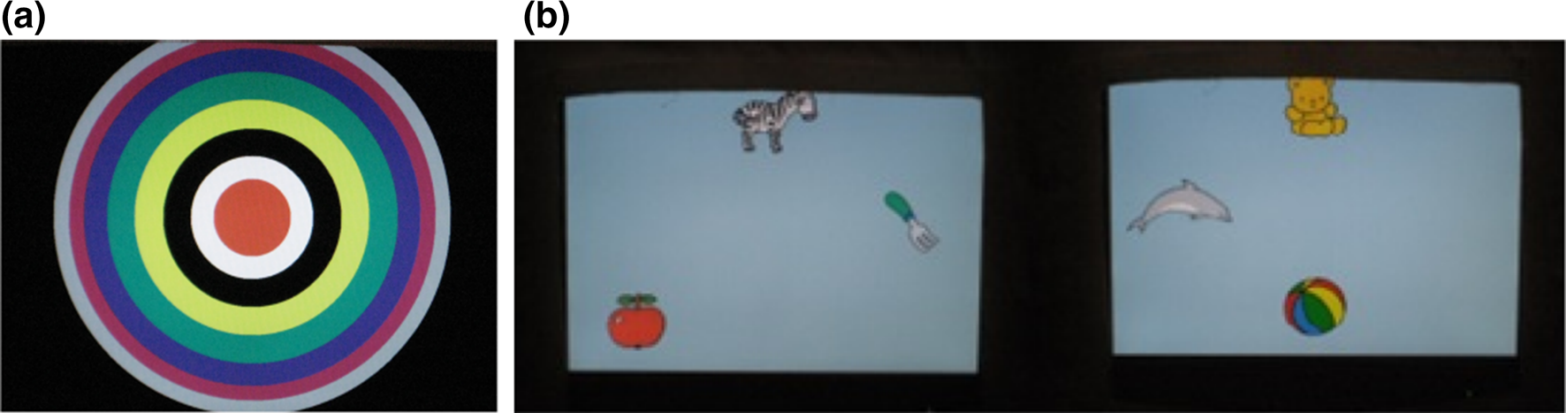
**a** Visual stimulus in the habituation, test and post-test phases (*left*). **b** Visual stimuli in the song phase (*right*)

Infants sat on their caretakers’ lap in the test booth, facing the screen (15” monitor Philips LCD 150P4) and the hidden camera (Colour video camera JVC TK-C1481EG) during the experiment. No visual or auditory distractions were present in the booth. An experimenter observed infants through a closed-circuit TV (Sony Trinitron KV-21T1D) in a room adjacent to the test booth. The infants went through three phases during the experiment: habituation, test, and post-test. The sound source (Tannoy X speaker) was placed behind the screen in the test booth. Repeated tokens of one tone were provided in the habituation phase. The test phase began when the mean looking time of the last three trials in the habituation phase fell below 65% of the mean looking time of the first three trials. Two trials of tokens of the other tone were presented in the test phase. In the post-test phase, a novel stimulus was presented to verify infants’ general attention, followed by a children’s song at the end to boost infants’ pleasure in participating the experiment. During the experiment, the dependent variable was infant looking time. The length of each trial was controlled by infant gazing: one trial ended when the infant looked away for more than 2 s, and then the next trial began. The inter-stimulus interval was set at one second in all phases. Discrimination was indicated by looking time rebound upon hearing the new stimulus during the phase change (from habituation to test phase). Infants’ looking time was recorded using a button box (two buttons). The stimuli in habituation and test phases are counterbalanced. The entire test was run via a computer program (ZEP, Veenker 2013). The visual stimuli were static bull’s eye in the habituation, test, and post-test phases (Fig. 3a), and random toy pictures appearing on a 3*3 grid when the children’s song was played (Fig. 3b). Caregivers were blind to the purpose of the test as well as the acoustic stimuli presented to infants throughout the experiment (wearing Headphones Echelon Telex).

## Results

**Fig. 4:**
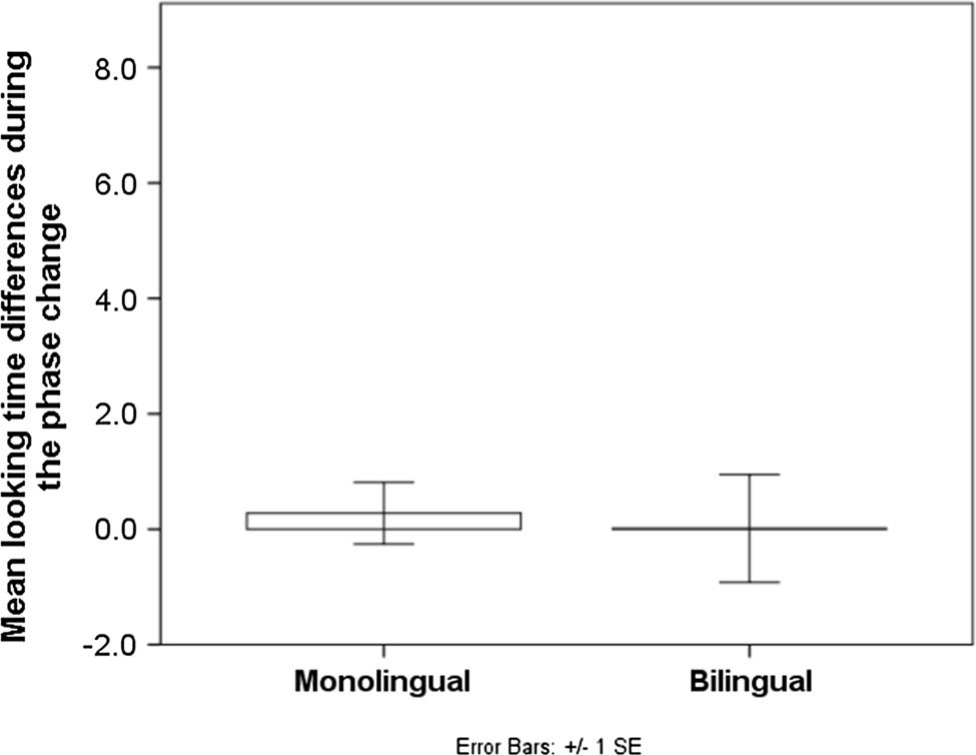
Mean looking time differences during the phase change

Infants’ mean looking times between the last two habituation trials and the two test trials were compared using a repeated measures analysis of variance (RM ANOVA). The between-subject factor was language background (2-level, monolingual versus bilingual). The main effect of the phase change (the difference between the two last trials in the habituation phase and the two trials in the test phase) was not significant, *F* (1, 34) = 0.073, *p* = 0.789, *η* ^2^ = 0.002. Neither was the interaction between language background and phase change, *F* (1, 34) = 0.061, *p* = 0.806, *η* ^2^ = 0.002. Hence, infants in both language backgrounds failed to discriminate the contrast (Fig. 4). Additionally, the effects of habituation order (*p* = 0.744) and second L1 differences (see Appendix, *p* = 0.990) were not significant, although the latter could be due to the diverse language backgrounds of the participating families.

## Discussion

Sensitivity to native and even non-native lexical pitch contrasts is maintained in tone-learning infants at 9 months (Yeung et al. 2013). Growing up in a non-tone language environment, Dutch infants no longer show sensitivity to the lexical pitch contrast at 8–9 months, conforming to previous findings on 8- to 9-month-old English and French infants (Mattock and Burhman 2006; Mattock et al. 2008). We interpret the lack of discrimination as due to language-specific perceptual attunement taking place in the first year after birth. In other words, infants may follow the “use-it-or-lose-it” strategy in the course of native phonemic category establishment. Alternatively, the current data can be interpreted as a “floor effect”. That is, the contrast is too difficult for infants to discriminate. The contrasted pitch, for instance, may sound natural only to native ears. Nevertheless, we believe this explanation is unlikely. Albeit lacking the ability to discriminate the same contrast, Dutch adults reported that they could hear the Mandarin lexical pitch, but were confused by the pitch direction (Liu et al., 2016). Meanwhile, non-tone language learning infants of 5–6 months are sensitive to the same contrast (Liu and Kager 2014), indicating perceptual attunement of lexical pitch.

Crucially, our data suggest that infant performance does not vary with their linguistic experience. When both languages are non-tonal, bilingual infants’ discrimination patterns match those of their monolingual peers. Unlike some previous findings, neither a perceptual delay nor an acceleration effect was observed among bilingual infants. Language-specific perceptual attunement appears to affect monolingual and bilingual infants equally. The fact that bilingual exposure does not alter the attunement process indicates that (1) infant sensitivity to lexical pitch is experience dependent; and (2) maturational factors may play a role in the perceptual attunement process apart from input-dependent factors.

## Experiment 2

Experiment 1 tested linguistic pitch processing with results showing no discrimination among Dutch monolingual and bilingual infants. To understand infants’ pitch processing across domains, a musical pitch contrast was examined in Experiment 2. Similar outcomes between the two experiments would be expected if the same perceptual mechanism underlies infant language and music perception, whereas any perceptual differences may indicate otherwise.

### Participant

Forty-eight Dutch monolingual and bilingual infants aged 9 months participated in Experiment 2. To prevent potential perceptual biases introduced by the same F0 among stimuli in the two experiments, all infants were different from those in Experiment 1. The same infant selection criteria as in Experiment 1 were adopted (see “Appendix” for bilingual language background information). The mean (SD) degree of exposure to Dutch was 53% (16%) among bilingual infants. Eventually, data from 36 participants were included in analysis, with 18 participants per language background (mean age (SD): 268(16) days; 50% males). Data of 12 infants from the initial sample pool were excluded from analyses for the following reasons: tone or pitch accent language exposure after birth (3), fussiness (3), crying (3), unable to habituate (1), and inattentiveness (2).

### Stimuli

**Fig. 5:**
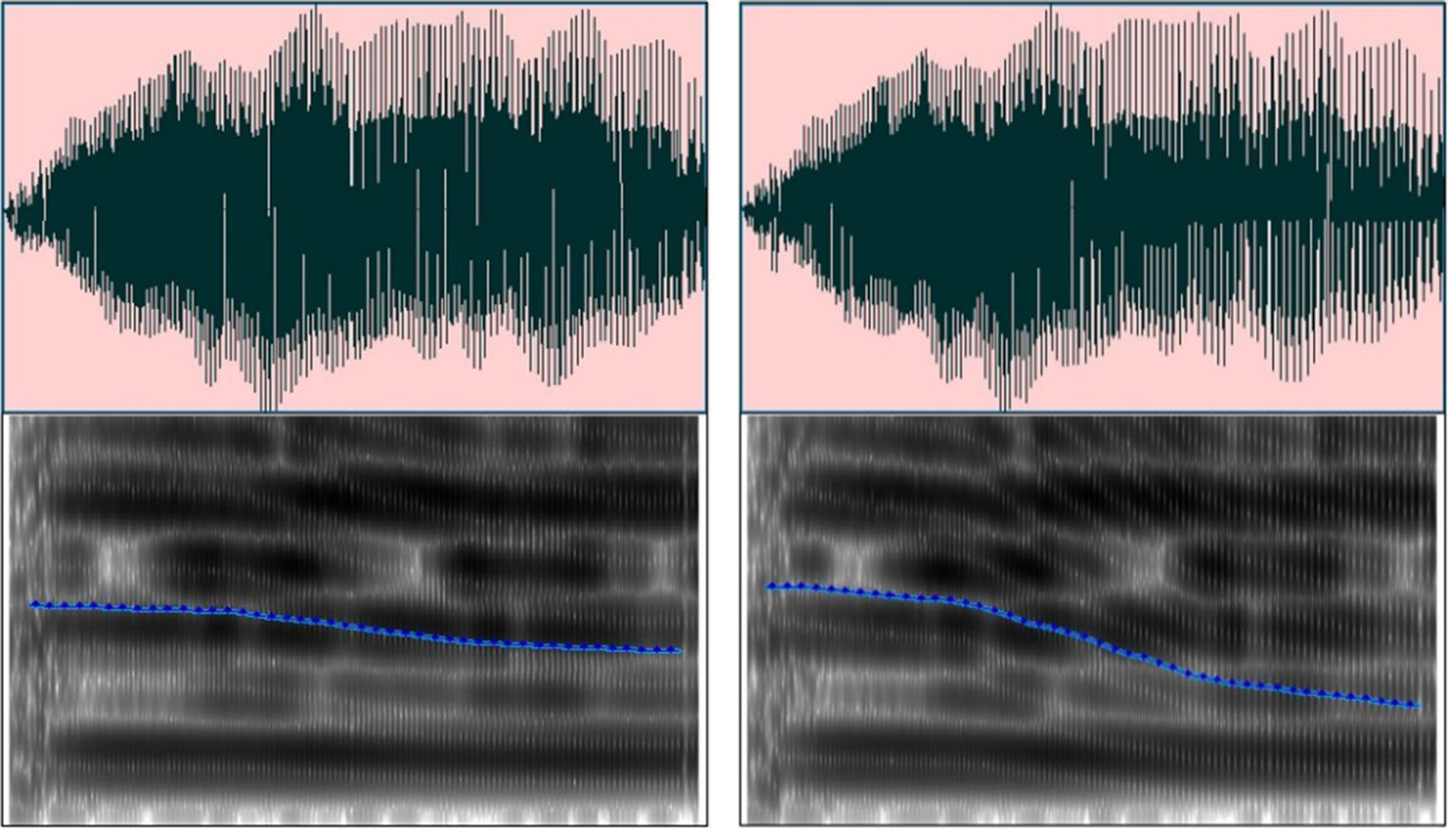
Oscillograms and spectrograms for the violin contrast

To ensure the cross-domain comparison, the musical (violin) tonal stimuli were generated from the same contrast used in Experiment 1. The F0 tiers of the contrasted tonal contrast in Experiment 1 were extracted and replaced the F0 tiers of a violin tone via PRAAT, creating novel violin stimuli. In other words, the violin contrast shared the exact same pitch contour as the tonal contrast in Experiment 1, but differed in timbre (Fig. 5). Four violin pairs were generated, matching the stimuli design in the first experiment. Five musicians listened to the stimuli and judged that they sounded natural. Musical pitch carried by a single note has seldom been tested. Even though the largest difference (50 Hz) along the contrast was within infants’ range of acoustic detection threshold (larger than one semitone within the range between 440 and 880 Hz), the overall acoustic differences may be too small to detect, resulting in non-discrimination.

### Procedure

The same procedure as in Experiment 1 was adopted.

## Results

**Fig. 6:**
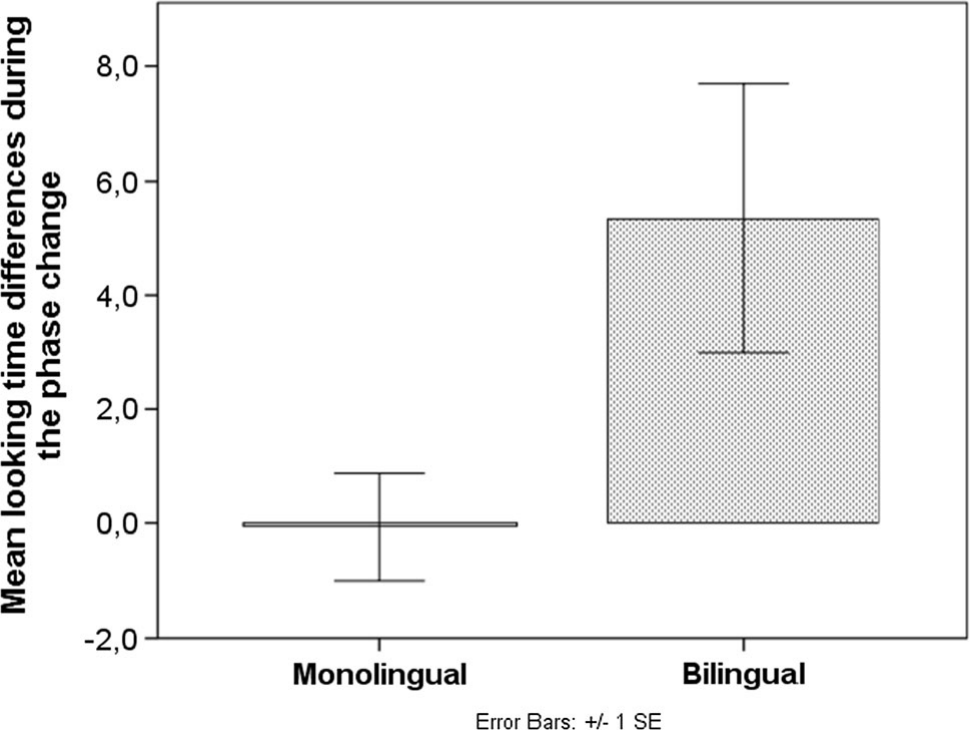
Mean looking time differences during the phase change

Infants’ mean looking times between the last two habituation trials and the two test trials were compared using an RM ANOVA. The between-subject factor was language background (2-level, monolingual versus bilingual). The main effect of the phase change was significant, *F* (1, 34) = 4.371, *p* = 0.044, *η* ^2^ = 0.114. The interaction between language background and the phase change was also significant, *F* (1, 34) = 4.565, *p* = 0.040, *η* ^2^ = 0.118. Splitting the data by language background, paired samples *t*-test shows that the phase change was not significant for the monolingual group, *t* (17) = 0.062, *p* = 0.951. The bilingual group presented significant looking time recovery, *t* (17) = −2.274, *p* = 0.036. Hence, bilingual but not monolingual infants discriminated the contrast (Fig. 6). Additionally, the effects of habituation order (*p* = 0.276) and second L1 differences (see Appendix, *p* = 0.224) were not significant, although the latter could be due to the high diversity of language backgrounds.

Pooling the current data with the ones in Experiment 1, an RM ANOVA was conducted with pitch type (2-level, linguistic versus musical) as an additional between-subject factor. The main effect of the phase change was significant, *F* (1, 68) = 4.118, *p* = 0.046, *η* ^2^ = 0.057. The interaction between language background and the phase change revealed a trend, *F* (1, 68) = 3.493, *p* = 0.066, *η* ^2^ = 0.049, as did the interaction between pitch type and phase change, *F* (1, 68) = 3.304, *p* = 0.073, *η* ^2^ = 0.046. The interaction across language background, pitch type, and the phase change was significant, *F* (1, 68) = 4.254, *p* = 0.033, *η* ^2^ = 0.059. Splitting the data by language background, an RM ANOVA showed that neither the phase change (*F* (1, 34) = 0.042, *p* = 0.839, *η* ^2^ = 0.001) nor the interaction between pitch type and the phase change (*F* (1, 34) = 0.098, *p* = 0.756, *η* ^2^ = 0.003) was significant for the monolingual group. In the bilingual group, however, both the phase change (*F* (1, 34) = 4.486, *p* = 0.042, *η* ^2^ = 0.117) and the interaction between pitch type and the phase change (*F* (1, 34) = 4.445, *p* = 0.042, *η* ^2^ = 0.116) were significant. In other words, 9-month-old monolingual infants did not discriminate the musical pitch contrast, whereas their bilingual peers succeeded.

## Discussion

Infants are predisposed to attending to musical melodies upon birth, showing sensitivity to musical pitch (Perani et al. 2010) just as they are sensitive to linguistic pitch regardless of language backgrounds (Nazzi et al. 1998). Nevertheless, 9-month-old Dutch monolingual infants failed to show discrimination in the current experiment. This is likely to be due to the acoustic properties of the stimuli, as single notes (less than 500 ms per token) were used with the sole difference lying in the final drop in pitch (50 Hz difference in the final part) between the two tokens. The outcomes point to a contrast-dependent nature of human music perception, which further indicates that the influence of acoustic salience applies to both language and music perception.

The most interesting finding of the current study is that unlike their monolingual peers, bilingual infants discriminated the musical contrast. This is unlikely to be due to attentional or memory factors (Singh et al. 2015) since (1) bilinguals did not perform better when perceiving a similar contrast in Experiment 1; (2) all infants included in the analysis passed the attention criterion; and (3) the cognitive load of the specific habituation paradigm is relatively low. Following infants’ advantage in native vowel and non-native tone discrimination (Liu and Kager 2016 in press), we propose a heightened acoustic sensitivity hypothesis among bilingual infants: facing a more complicated learning environment, bilinguals may be more sensitive to the subtle acoustic differences in the incoming stimuli, and this sensitivity is not restricted to speech contrasts but extends to the music domain. It is surprising that bilingual infants discriminated the violin tonal contrast more than the linguistic tonal contrast. We discuss possible explanations and implications in the next section.

## General discussion

Dutch monolingual and bilingual infants of 8–9 months were tested on a linguistic and a musical pitch contrast. In the perception of lexical pitch, no discrimination was found in Dutch monolingual and bilingual infants. A tentative explanation might be that the contrast may be too difficult for infants at this age. This interpretation, however, is not in line with previous findings reporting initial sensitivity to pitch in neonates (Nazzi et al. 1998), younger Dutch infants’ sensitivity to the same contrast (5–6 months, Liu and Kager 2014), and older Dutch infants’ sensitivity to the same contrast when they are exposed to a statistical frequency distribution that favours learning (11–12 months, Liu and Kager 2011; under review). Alternatively, non-tone language learning infants may pay little attention to non-native pitch at the end of the perceptual attunement period, since lexical pitch does not contrast word meaning in their native language inventory. The lack of sensitivity to non-native linguistic pitch reported for infants across language backgrounds (e.g. Mattock and Burnham 2006) is presumably due to the lack of exposure to a tone language. These findings suggest that input is a key factor underlying language-specific perceptual attunement. Music-wise, albeit infants’ initial sensitivity, Dutch monolingual infants did not discriminate the current violin contrast. Their perceptual patterns to musical pitch appear to vary as a function of acoustic salience (Trainor and Trehub 1992; Trehub et al. 1997), similar to the influence of acoustic salience in language (Liu and Kager 2015b).

Crucially, bilingual infants outperformed their monolingual peers when perceiving the violin pitch contrast, illustrating bilingual perceptual enhancement in the music domain. This novel finding is unlikely to be due to different exposure as the hours of exposition to music at home were comparable in the two groups from parental feedback. It is unlikely that bilingual infants have a systematically longer exposure than their monolingual peers. The systematic exposure to a second L1 nevertheless leads to greater acoustic sensitivity to the musical contrast even when both languages are not tonal. Accordingly, we interpret the current results as a domain-general effect stemming from a bilingual environment. Previous studies demonstrate cognitive gains among bilingual infants as early as 7 months (e.g. inhibition control; Kovács and Mehler 2009a, b). We hypothesize that bilingual infants present another cognitive advantage, heightened acoustic sensitivity, compared to their monolingual peers (Liu 2014). Specifically, bilinguals may focus on input acoustic details more than their monolingual peers. Heightened acoustic sensitivity has been shown in bilingual infants in the linguistic domain in the first year after birth. Bilingual infants of 8–9 months acquire a native vowel contrast, 3 months earlier than monolinguals (Liu and Kager 2016a). Bilingual infants of 11–12 months recover their sensitivity to lexical pitch, 6 months earlier than monolinguals (Liu and Kager 2016b). We hypothesize that this advantage, resulting from a bilingual environment, applies to acoustic perception across language and music domains. Furthermore, the cross-domain effect indicates that such heightened sensitivity may be acoustic rather than linguistic in nature.

Although Dutch monolingual infants of 8–9 months show similar perceptual patterns between lexical and musical contrasts, the patterns differ in bilingual population. What may be the explanations of the discrepancies in the discrimination of the two contrasts among bilingual infants? Since F0 remains constant between the lexical and the musical pitch contrast, the other formants and acoustic properties must play important roles in speech and music processing. One possibility is that the non-native linguistic pitch contrast is processed acoustically by Dutch infants as early as 9 months, while the acoustic salience of the linguistic pitch contrast is somehow lower than the violin contrast. This possibility is not implausible, although if true, it is unclear why bilingual infants are more drawn to the violin contrast. We hypothesize that musical contrasts are typically more consistent in overall formant production, making it easier for infants to capture the F0 differences. Nevertheless, one can argue the other way around and claim that the inconsistency in linguistic pitch contrast may provide extra acoustic cues to infants and/or attract more of their attention. Additionally, previous research has shown that infants prefer human voice to other acoustically complex stimuli (Siperstein and Butterfield 1972; DeCasper and Fifer 1980). We leave this possibility open for future research.

An alternative explanation would be that infants’ different perceptual patterns may indicate early processing diversion between language and music. This hypothesis leads to the discussion and debate on how the two domains interact. Earlier studies show autonomy between language and music processing. Newborn infants’ hemispheric dominances differ between language (left hemisphere, Witelson and Pallie 1973) and music (right hemisphere, Balaban et al. 1998). Individuals with a disorder in one domain may have the other domain largely intact (Ullman et al. 1997; Hébert et al. 2003; Peretz et al. 2003; Peretz and Coltheart 2003; Racette et al. 2006; Wilson et al. 2006; Schlaug et al. 2008). The majority of tone language (lexical pitch variations distinguish meanings) speakers with amusia are able to perceive and produce their native tones accurately (Nan et al. 2010). Neural studies have pinpointed specific areas involved in language and music processing (Fedorenko et al. 2011). Nevertheless, recent research reports a trend of interdependence between language and music. Both musicianship and speaking a tone language appear to be mutually beneficial in the complementary domain. Non-tone language listeners’ detection of lexical tonal variations is related to their music aptitude and melodic ability. Non-tone language musicians are more accurate than non-musicians when perceiving pitch, and this improvement is transferred to the perception of lexical tones (Alexander et al. 2005; Delogu et al. 2006; Marie et al. 2011). Cantonese and Chinese (tone language) listeners outperform Canadian French, Dutch, and English (non-tone language) listeners in melodic discrimination abilities and musical pitch perception (Bidelman et al. 2013; Chen et al. 2015). French amusic listeners illustrate impaired perception of lexical tones and the non-speech analogues of the tones (Tillmann et al. 2011). Chinese or Cantonese amusic listeners present deficits when processing their native intonation (Jiang et al. 2010), yet they are more accurate when perceiving musical pitch than their Canadian French and English counterparts (Wong et al. 2012). When trained to use pitch patterns to differentiate meanings of pseudo-English words, English listeners’ learning successes are associated with participants’ sensitivity to pitch in a non-linguistic context as well as their previous musical experience (Wong and Perrachione 2007).

Unlike tone language adult listeners who perceive linguistic pitch categorically, non-tone language adult listeners perceive linguistic pitch in a psycho-acoustic manner (Hallé et al. 2004; Xu et al. 2006). The neural responses of non-native pitch are in accordance with musical pitch, indicating that non-tone language adult listeners do not treat lexical pitch as linguistically relevant (Francis et al. 2003). Since the perception of musical pitch and lexical pitch are psycho-acoustically driven (Delogu et al. 2006; Marie et al. 2011; Tillmann et al. 2011), it is reasonable to expect that the two domains are unified for non-tone language learning adult listeners. It remains unclear whether the same patterns hold for non-tone language learning infants.

The current study focuses on the perception of pitch by non-tone language learning infants and seeks to determine whether cross-domain (dis)association may occur during the first year after birth. Our findings appear to support dissociation between language and music in the first year after birth, in line with previous literature reporting early dispersion crossing the two domains (Fedorenko et al. 2011). Although research on this topic is still at an immature stage where various studies lead to opposite conclusions, our data suggest that the two domains coexist in a bimodular fashion, though not without interaction since an influence of linguistic exposure was observed on a musical pitch perception task.

Several frameworks have been proposed to account for previous and current findings of dissociations and similar processing mechanism between language and music (Patel 2013; Patel et al. 2008; Peretz 2006). These frameworks posit that language and music share closely related cortical processing and functional computation mechanisms yet can be dissociated by neurological abnormalities. Furthermore, the current study leads to the discussion of the origin and development between language and music. The possible early diversion between language and music may suggest that the human brain is equipped with music-specific neural networks, evidenced by congenital amusic patients. The claim that music might be distinct from other cognitive functions (Peretz and Hyde 2003) indicates that human musical abilities, just as those involved in language, should not be simply considered as an evolutionary by-product of other cognitive functions.

Finally, we address some areas that are out of the scope of the current study. First, the current study adopts behavioural measures for infant language and music processing. Infants’ neural responses when perceiving the same stimuli need to be investigated in order to identify the nature of (linguistic versus psycho-acoustic) processing. The early trace and localization of cross-domain perceptual diversion and the difference across infants from different language backgrounds can be studied using brain-imaging techniques. Second, it has been hypothesized that the cognitive benefit of learning lexical tones—enhanced pitch sensitivity—may persist into adulthood and diffuse to musical pitch processing by tone language listeners (Chen et al. 2016). Through acquiring the lexically contrastive function of pitch, tone language listeners become more accurate in perceiving pitch acoustics and this higher accuracy is transferred to the music domain (Wong et al. 2012; Bidelman et al. 2013). The language and music pitch perception of tone language learning infants needs to be verified. Third, to understand the scope of the bilingual influence in music perception observed in the current study, both infant and adult listeners should be tested across language backgrounds (Liu et al. in preparation). Fourth, the F0 in the current study is kept constant across linguistic and musical stimuli. Since tone language adult listeners illustrate positive transfer effect when perceiving pitch changes across the two domains, it remains unclear whether such transfer effect would remain when pitch information is changed among infants. The answer will be revealed with future data from tone or pitch accent language learning infants. Fifth, the current study tested one lexical tone and one violin tone contrast. Different musical tonal pairs need to be investigated to understand the effect of acoustic salience in the musical domain. Sixth, the current paper reports infants exposed to multiple languages are better at discriminating violin pitch contrasts. It remains unclear whether extensive music experience would facilitate the perception of linguistic pitch. This question helps understand the scope of the cross-domain interaction. Last but not least, questions emerge when comparing similarities between the rhythmic classes of language and acoustic properties of music. For examples, does (enhanced) exposure to a syllable-timed language lead to enhanced musical rhythm perception? Does exposure to a stress-timed language promote the perception of meter? Does additional exposure to a tone language sharpen one’s perception of contour, scale, and interval? It has been shown that spoken prosody may leave an imprint on the music of a culture (Patel and Daniele 2003). We leave these questions open for future research.

## Appendix

See Table 1.

**Table 1:** Bilingual language background apart from Dutch

	Experiment 1	Experiment 2
Afrikaans	1	0
Czech	1	0
English	5	2
French	1	1
Frisian	1	0
German	4	6
Hebrew	1	0
Italian	1	1
Portuguese	0	1
Russian	0	3
Spanish	2	3
Turkish	1	1
Total	18	18

## Footnotes

1 Audacity open resource: ‘http://audacity.sourceforge.net’.

## References

[CR1] Albareda-Castellot B, Pons F, Sebastián-Gallés N (2011). The acquisition of phonetic categories in bilingual infants: New data from an anticipatory eye movement paradigm. Dev Sci.

[CR2] Alexander JA, Wong PC, Bradlow AR (2005) Lexical tone perception in musicians and non-musicians. Interspeech 397–400.

[CR3] Balaban MT, Anderson LM, Wisniewski AB (1998). Lateral asymmetries in infant melody perception. Dev Psychol.

[CR4] Bidelman GM, Hutka S, Moreno S (2013). Tone language speakers and musicians share enhanced perceptual and cognitive abilities for musical pitch: Evidence for bidirectionality between the domains of language and music. PLoS One.

[CR5] Boersma P, Weenink D (2010) {P} raat: Doing phonetics by computer

[CR6] Bosch L, Sebastián-Gallés N (2001). Evidence of early language discrimination abilities in infants from bilingual environments. Infancy.

[CR7] Bosch L, Sebastián-Gallés N (2003). Simultaneous bilingualism and the perception of a language-specific vowel contrast in the first year of life. Lang Speech.

[CR8] Brito N, Barr R (2012). Influence of bilingualism on memory generalization during infancy. Dev Sci.

[CR9] Brito N, Barr R (2014). Flexible memory retrieval in bilingual 6-month-old infants. Dev Psychobiol.

[CR10] Burns TC, Yoshida KA, Hill K, Werker JF (2007). The development of phonetic representation in bilingual and monolingual infants. Appl Psycholinguist.

[CR11] Chang HW, Trehub SE (1977) Infants’ perception of temporal grouping in auditory patterns. Child Dev 1666–1670. 608377

[CR12] Chen A, Liu L, Kager R (2015). Cross-linguistic perception of Mandarin tone sandhi. Lang Sci.

[CR13] Chen A, Liu L, Kager R (2016). Cross-domain correlation in pitch perception, the influence of native language. Lang Cognit Neurosci.

[CR14] DeCasper AJ, Fifer WP (1980). Of human bonding: Newborns prefer their mothers’ voices. Science.

[CR15] Delogu F, Lampis G, Belardinelli MO (2006). Music-to-language transfer effect: May melodic ability improve learning of tonal languages by native nontonal speakers?. Cognit Process.

[CR16] Dietrich C, Swingley D, Werker JF (2007). Native language governs interpretation of salient speech sound differences at 18 months. Proc Natl Acad Sci.

[CR17] Fedorenko E, Behr MK, Kanwisher N (2011). Functional specificity for high-level linguistic processing in the human brain. Proc Natl Acad Sci.

[CR18] Fernald A (1991). Prosody in speech to children: Prelinguistic and linguistic functions. Ann Child Dev.

[CR19] Francis AL, Ciocca V, Ng BKC (2003). On the (non) categorical perception of lexical tones. Percept Psychophys.

[CR20] Garcia-Sierra A, Rivera-Gaxiola M, Percaccio CR, Conboy BT, Romo H, Klarman L (2011). Bilingual language learning: An ERP study relating early brain responses to speech, language input, and later word production. J Phon.

[CR21] Gauthier K, Genesee F (2011). Language development in internationally adopted children: A special case of early second language learning. Child Dev.

[CR22] Hallé PA, Chang YC, Best CT (2004). Identification and discrimination of Mandarin Chinese tones by Mandarin Chinese vs. French listeners. J Phon.

[CR23] Harrison P (2000). Acquiring the phonology of lexical tone in infancy. Lingua.

[CR24] Hauser MD, Chomsky N, Fitch WT (2002). The faculty of language: What is it, who has it, and how did it evolve?. Science.

[CR25] Hébert S, Racette A, Gagnon L, Peretz I (2003). Revisiting the dissociation between singing and speaking in expressive aphasia. Brain.

[CR26] Huang T, Johnson K (2010). Language specificity in speech perception: Perception of Mandarin tones by native and nonnative listeners. Phonetica.

[CR27] Jiang C, Hamm JP, Lim VK, Kirk IJ, Yang Y (2010). Processing melodic contour and speech intonation in congenital amusics with Mandarin Chinese. Neuropsychologia.

[CR28] Kovács ÁM, Mehler J (2009). Flexible learning of multiple speech structures in bilingual infants. Science.

[CR29] Kovács ÁM, Mehler J (2009). Cognitive gains in 7-month-old bilingual infants. Proc Natl Acad Sci.

[CR30] Kuhl PK, Williams KA, Lacerda F, Stevens KN, Lindblom B (1992). Linguistic experience alters phonetic perception in infants by 6 months of age. Science.

[CR31] Kuhl PK, Conboy BT, Coffey-Corina S, Padden D, Rivera-Gaxiola M, Nelson T (2008). Phonetic learning as a pathway to language: New data and native language magnet theory expanded (NLM-e). Philos Trans R Soc B Biol Sci.

[CR32] Kuipers JR, Thierry G (2012). Event-related potential correlates of language change detection in bilingual toddlers. Dev Cognit Neurosci.

[CR33] Kuipers JR, Thierry G (2013). ERP-pupil size correlations reveal how bilingualism enhances cognitive flexibility. Cortex.

[CR34] Liu L (2014). The effects of bilingualism on infant language development: the acquisition of sounds and words.

[CR35] Liu L, Kager R (2011) Is perceptual reorganization affected by statistical learning? Dutch infants’ sensitivity to lexical tones. In: Proceedings of the 35th annual Boston University conference on language development, Cascadilla Press, Somerville, pp. 404–413

[CR36] Liu L, Kager R (2014). Perception of tones by infants learning a non-tone language. Cognition.

[CR37] Liu L, Kager R (2015). Bilingual exposure influences infant VOT perception. Infant Behav Dev.

[CR38] Liu L, Kager R (2015). Understanding phonological acquisition through phonetic perception: The influence of exposure and acoustic salience. Phonol Stud.

[CR39] Liu L, Kager R (2016a) Perception of a native vowel contrast by Dutch monolingual and bilingual infants: a bilingual perceptual lead. Int J Billing 1367006914566082.

[CR40] Liu L, Kager R (2016). Perception of tones by bilingual infants learning non-tone languages. Biling Lang Cognit.

[CR41] Liu, L. & Kager, R. (in press). Is mommy talking to daddy or to me? Exploring parental estimates of child language exposure using the Multilingual Infant Language Questionnaire. Int J Billing. http://dx.doi.org/10.1080/14790718.2016.1216120.

[CR42] Liu L, Kager R (under review). Dutch infants’ lexical tone perception under distributional learning. J Chile Exp Psychol

[CR43] Liu L, Chen A, Kager R (2016) Tone perception in Mandarin and Dutch adult listeners. Lang Linguist

[CR44] Lynch MP, Eilers RE (1992). A study of perceptual development for musical tuning. Percept Psychophys.

[CR45] Marie C, Delogu F, Lampis G, Belardinelli MO, Besson M (2011). Influence of musical expertise on segmental and tonal processing in Mandarin Chinese. J Cognit Neurosci.

[CR46] Mattock K, Burnham D (2006). Chinese and English infants’ tone perception: Evidence for perceptual reorganization. Infancy.

[CR47] Mattock K, Molnar M, Polka L, Burnham D (2008). The developmental course of lexical tone perception in the first year of life. Cognition.

[CR48] McDermott JH, Oxenham AJ (2008). Music perception, pitch, and the auditory system. Curr Opin Neurobiol.

[CR49] Nan Y, Sun Y, Peretz I (2010) Congenital amusia in speakers of a tone language: association with lexical tone agnosia. Brain, awq178 10.1093/brain/awq178 20685803

[CR50] Nazzi T, Floccia C, Bertoncini J (1998). Discrimination of pitch contours by neonates. Infant Behav Dev.

[CR51] Patel, A. D. (2013). Sharing and nonsharing of brain resources for language and music. In: Michael, A. Arbib (ed) From Language, music, and the brain, Strüngmann Forum Reports, vol. 10, J. Lupp, series ed. Cambridge, MA: MIT Press

[CR52] Patel AD, Daniele JR (2003). An empirical comparison of rhythm in language and music. Cognition.

[CR53] Patel AD, Iversen JR, Wassenaar M, Hagoort P (2008). Musical syntactic processing in agrammatic Broca’s aphasia. Aphasiology.

[CR54] Perani D, Saccuman MC, Scifo P, Spada D, Andreolli G, Rovelli R (2010). Functional specializations for music processing in the human newborn brain. Proc Natl Acad Sci.

[CR55] Peretz I (2006). The nature of music from a biological perspective. Cognition.

[CR56] Peretz I, Coltheart M (2003). Modularity of music processing. Nature Neurosci.

[CR57] Peretz I, Hyde KL (2003). What is specific to music processing? Insights from congenital amusia. Trends Cognit Sci.

[CR58] Peretz I, Champod AS, Hyde K (2003). Varieties of musical disorders. Ann N Y Acad Sciences.

[CR59] Petitto LA, Berens MS, Kovelman I, Dubins MH, Jasinska K, Shalinsky M (2012). The “Perceptual Wedge Hypothesis” as the basis for bilingual babies’ phonetic processing advantage: New insights from fNIRS brain imaging. Brain Lang.

[CR60] Racette A, Bard C, Peretz I (2006). Making non-fluent aphasics speak: Sing along!. Brain.

[CR61] Schellenberg EG, Trehub SE (1999). Culture-general and culture-specific factors in the discrimination of melodies. J Exp Child Psychol.

[CR62] Schlaug G, Marchina S, Norton A (2008). From singing to speaking: Why singing may lead to recovery of expressive language function in patients with Broca’s aphasia. Music Percept Interdiscip J.

[CR63] Sebastián-Gallés N, Bosch L (2009). Developmental shift in the discrimination of vowel contrasts in bilingual infants: Is the distributional account all there is to it?. Dev Sci.

[CR64] Shafer VL, Yan HY, Datta H (2011). The development of English vowel perception in monolingual and bilingual infants: Neurophysiological correlates. J Phonet.

[CR65] Singh L, Fu CS, Rahman AA, Hameed WB, Sanmugam S, Agarwal P, Jiang B, Chong YS, Meaney MJ, Rifkin-Graboi A, GUSTO Research Team (2015). Back to basics: a bilingual advantage in infant visual habituation. Child Dev.

[CR66] Siperstein GN, Butterfield EC (1972) Neonates prefer vocal-instrumental music to noise and vocal music to instrumental music. In: Oral sensation and perception. Springfield: Chas. Thomas

[CR67] Sun KC, Huang T (2012). A cross-linguistic study of Taiwanese tone perception by Taiwanese and English listeners. J East Asian Ling.

[CR68] Sundara M, Scutellaro A (2011). Rhythmic distance between languages affects the development of speech perception in bilingual infants. J Phonet.

[CR69] Sundara M, Polka L, Molnar M (2008). Development of coronal stop perception: Bilingual infants keep pace with their monolingual peers. Cognition.

[CR70] Tillmann, B., Burnham, D., Nguyen, S., Grimault, N., Gosselin, N., & Peretz, I. (2011). Congenital amusia (or tone-deafness) interferes with pitch processing in tone languages. Front Psychol, 2 10.3389/fpsyg.2011.00120 PMC3119887 21734894

[CR71] Trainor LJ, Trehub SE (1992). A comparison of infants’ and adults’ sensitivity to western musical structure. J Exp Psychol Hum Percept Perform.

[CR72] Trehub SE, Hannon EE (2006). Infant music perception: Domain-general or domain-specific mechanisms?. Cognition.

[CR73] Trehub SE, Bull D, Thorpe LA (1984) Infants’ perception of melodies: the role of melodic contour. Child Dev 821–830 10.1111/j.1467-8624.1984.tb03819.x 6734320

[CR74] Trehub SE, Thorpe LA, Morrongiello BA (1985). Infants’ perception of melodies: Changes in a single tone. Infant Behav Dev.

[CR75] Trehub S, Schellenberg EG, Hill DS (1997) The origins of music perception and cognition: a developmental perspective

[CR76] Ullman MT, Corkin S, Coppola M, Hickok G, Growdon JH, Koroshetz WJ, Pinker S (1997). A neural dissociation within language: Evidence that the mental dictionary is part of declarative memory, and that grammatical rules are processed by the procedural system. J Cognit Neurosci.

[CR77] Veenker T (2013). The ZEP experiment control application.

[CR78] Wang Y, Jongman A, Sereno JA (2001). Dichotic perception of Mandarin tones by Chinese and American listeners. Brain Lang.

[CR79] Werker J (2012). Perceptual foundations of bilingual acquisition in infancy. Ann N Y Acad Sci.

[CR80] Werker JF, Tees RC (1984). Cross-language speech perception: Evidence for perceptual reorganization during the first year of life. Infant Behav Dev.

[CR81] Werker JF, Tees RC (2005). Speech perception as a window for understanding plasticity and commitment in language systems of the brain. Dev Psychobiol.

[CR82] Wilson SJ, Parsons K, Reutens DC (2006) Preserved singing in aphasia

[CR83] Witelson SF, Pallie W (1973). Left hemisphere specialization for language in the newborn. Brain.

[CR84] Wong P, Perrachione TK (2007). Learning pitch patterns in lexical identification by native English-speaking adults. Appl Psycholinguist.

[CR85] Wong PC, Ciocca V, Chan AH, Ha LY, Tan LH, Peretz I (2012). Effects of culture on musical pitch perception. PLoS One.

[CR86] Xu Y, Gandour JT, Francis AL (2006). Effects of language experience and stimulus complexity on the categorical perception of pitch direction. J Acoust Soc Am.

[CR87] Yeung HH, Chen KH, Werker JF (2013). When does native language input affect phonetic perception? The precocious case of lexical tone. J Mem Lang.

